# Integrated Circuit Bonding Distance Inspection via Hierarchical Measurement Structure

**DOI:** 10.3390/s24123933

**Published:** 2024-06-18

**Authors:** Yuan Zhang, Chenghan Pu, Yanming Zhang, Muyuan Niu, Lifeng Hao, Jun Wang

**Affiliations:** 1 College of Mechanical and Electrical Engineering, Nanjing University of Aeronautics and Astronautics, Nanjing 210016, China; yuanzhang2020@nuaa.edu.cn (Y.Z.); wjun@nuaa.edu.cn (J.W.); 2 College of Computer Science and Technology, Nanjing University of Aeronautics and Astronautics, Nanjing 211106, China; niumuyuan@nuaa.edu.cn; 3 The 29th Research Institute of China Electronics Technology Group Corporation, Chengdu 610036, China; zhangyanming@cetc.com.cn (Y.Z.); npuhlf@126.com (L.H.)

**Keywords:** wire bonding, bonding distance, hierarchical measurement, bonding spot detection, gold wire segmentation

## Abstract

Bonding distance is defined by the projected distance on a substrate plane between two solder points of a bonding wire, which can directly affect the morphology of the bonding wire and the performance between internal components of the chip. For the inspection of the bonding distance, it is necessary to accurately recognize gold wires and solder points within the complex imagery of the chip. However, bonding wires at arbitrary angles and small-sized solder points are densely distributed across the complex background of bonding images. These characteristics pose challenges for conventional image detection and deep learning methods to effectively recognize and measure the bonding distances. In this paper, we present a novel method to measure bonding distance using a hierarchical measurement structure. First, we employ an image acquisition device to capture surface images of integrated circuits and use multi-layer convolution to coarsely locate the bonding region and remove redundant background. Second, we apply a multi-branch wire bonding inspection network for detecting bonding spots and segmenting gold wire. This network includes a fine location branch that utilizes low-level features to enhance detection accuracy for small bonding spots and a gold wire segmentation branch that incorporates an edge branch to effectively extract edge information. Finally, we use the bonding distance measurement module to develop four types of gold wire distribution models for bonding spot matching. Together, these modules create a fully automated method for measuring bonding distances in integrated circuits. The effectiveness of the proposed modules and overall framework has been validated through comprehensive experiments.

## 1. Introduction

**Figure 1:**
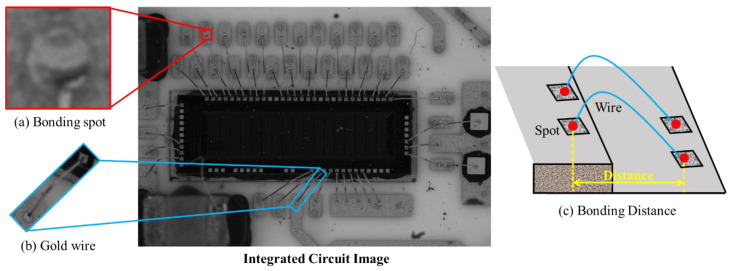
Example of an integrated circuit. (**a**) Zoomed-in view of wire bonding. The bonding distance is the projected distance between the centers of two bonding spots. (**b**) Image of an integrated circuit, with a resolution of 2448×2048. (**c**) Zoomed-in views of a bonding spot and a gold wire.

In the microelectronics industry, wire bonding forms an essential electrical connection in integrated circuits. The primary factors affecting bonding performance include wire defects, weak bonding spots, and abnormal gold wire morphology. The geometry of gold wires influences high-frequency communication performance between chips. Therefore, in practical engineering, it is necessary to ensure that both the gold wires and bonding points are free from anomalies. Additionally, the geometry of the gold wires needs to be inspected, with bonding distance being a crucial metric for assessing gold wire geometry. As shown in Figure 1, the bonding distance refers to the horizontal distance from the bond spot to the lead of the package substrate during the wire bonding process. This is the actual span of the gold wire from the chip pad to the substrate pin, not including the bent or height portion of the gold wire. In wire bonding inspection, the bonding distance is one of the most important indices. Abnormal distance, which is a typical defect, can lead to a short circuit or broken circuit. Specifically, if the bonding distance exceeds the value intended in the design of the circuit, the bonding spot will be beyond the circuit boundary, resulting in a broken circuit. Conversely, if the bonding distance is too short, the adjacent gold wires may come into contact and cause a short circuit. Therefore, the measurement of a wire bonding distance is very essential in ensuring the production quality of integrated circuits. Nevertheless, existing key distance measurement methods have some limitations. First of all, gold wires are small in size and complex in structure. In addition, given the complex background of wire bondings, it is difficult to extract them and measure bonding distances throughout an integrated circuit.

Methods currently in use for measuring and inspecting bonding distance include microscopy-based methods, two-dimensional (2D) image-based methods [1,2], and three-dimensional (3D) data-based methods [3]. Microscopy-based methods involve manual inspection of wire bondings one by one, which is a complex operation that requires a specialist worker. The second type of method involves inspecting the wire bonding based on a 2D image [1,2]. However, these approaches were designed for detecting wire bonding defects, such as breakage, loss, shifting, or sagging of the bonding, rather than for accurate bonding distance measurement. Although several state-of-the-art measurement methods [4,5] based on 2D image data have achieved good results recently in brake pad measurement and thread geometric error measurement, owing to the complex structure of gold wire bonding, simple dimensional measurement methods based on 2D images cannot be directly used for bonding distance measurement. The third type of approach involves measuring the bonding distance based on 3D point cloud data of the integrated circuit chips. Nonetheless, it is difficult to efficiently collect a 3D point cloud for gold wire bonding, as existing 3D scanning equipment cannot fully capture the necessary data for fine gold wire.

According to the above analysis, we believe that bonding distance measurement based on 2D images is practicable. In recent years, computer vision techniques [6,7,8,9], which represent an important research area in deep learning [10,11], have developed rapidly. Many computer-vision-based methods have been applied in various fields, including aero-engine blade defect detection [12], steel plate defect inspection [13], and visual measurements [14]. These methods all apply a fusion scheme to enhance feature learning ability and achieve state-of-the-art performance. Specifically, in wire bonding inspection, learning-based detection methods have achieved outstanding performance in solder spot detection, gold wire inspection, and other inspection tasks [15,16,17]. Therefore, the quality of wire bonding distance measurement could be improved by using learning-based methods to extract features of wire bonding.

However, because of the intricate structure and complex background of wire bonding, it is difficult to apply existing learning-based detection methods to accurately extract wire bonding, which in turn limits the accuracy of wire bonding distance measurements. For instance, the detailed information of small wire bonding objects is easily lost by classical convolutional neural network (CNN) detectors [18,19,20,21,22], as there are many pooling operations. Moreover, larger strides may also lead to information loss for small objects. In addition, a complex and redundant background increases the amount of computation and interferes with object detection.

To address these problems, we design a hierarchical inspection framework for the integrated circuit bonding distance inspection task. The proposed framework contains a multi-branch wire bonding inspection network and a bonding distance measurement module.

First, to remove noise and redundant background from integrated circuit images, we utilize a coarse location to extract the bonding region. Then, we develop the multi-branch wire bonding inspection network for bonding spot detection and gold wire segmentation on the extracted gold wire bonding regions of the images. Specifically, we use a learning-based fine location branch with a feature extraction module, a spatial correlation aware module, and a bidirectional feature fusion module. By establishing a feature extraction module based on dense blocks and dilated convolution operations, we can improve the extraction ability of bonding spots. In addition, we establish a spatial correlation aware module to extract high-level semantic information with a self-attention mechanism. As the gold wires and bonding spots have strong spatial correlations, this module can capture correlations among high-level feature entities. Moreover, we take advantage of the semantic information from the parallel segmentation branch to enhance the self-attention feature representation. Additionally, we employ a bidirectional feature extraction module in the fine location branch, which can effectively utilize low-level features to improve detection accuracy for small bonding spots, and a gold wire segmentation branch, including an edge branch, to extract edge information. The edge branch extracts the edge-related features of gold wire bonding via its gated convolution layer (GCL). Finally, we establish four types of gold wire distribution models to achieve bonding spot matching in the bonding distance measurement module (BDMM) with high accuracy. The centers of bonding spots are identified based on fitted circles, as all bonding spots have circular shapes. Therefore, we can confirm the bonding distance according to the identified center coordinates. Overall, the contributions of this study can be summarized as follows.

- We design a hierarchical measurement structure framework based on deep learning from 2D images to measure bonding distances in integrated circuits with high precision.
- We propose a novel multi-branch wire bonding inspection network (MWBINet) for wire bonding locations; each branch in the network provides auxiliary spatial correlation information for the others, which strongly enhances the feature representation, thereby solving the problem of limited target information when detecting very small targets.
- We propose the BDMM to implement bonding spot matching, thus achieving accurate bonding distance measurement.

Our experiments are exclusively based on integrated circuits without bonding point defects. This is due to the fact that the measurement of bonding distance is intended to identify potential short circuits or open circuits, which are vital for assessing the performance of the chip. If defects are present in the bonding points, the chip can be immediately classified as defective, eliminating the need for further measurement of the bond distance.

## 2. Related Work

### 2.1. Wire Bonding Inspection

As an essential electrical connection structure, wire bonding needs to be inspected during the production process of integrated circuits. Ko et al. [23] designed a system to measure the position of a wire during a wire bonding pull test, which can test the strength of wire efficiently. But this method is destructive and can only be used for sampling inspection. Feng et al. [1] proposed a method to monitor the quality of wire bonding by analyzing bonding voltage and current signals from an ultrasonic generator. However, these methods require contact and are difficult to operate, making them unsuitable for inspection during assembly-line production. Vision-based detection is an efficient non-contact detection method that has been widely studied with respect to its potential applications in the inspection of wire bonding. Perng et al. [2] proposed a system for wire bonding inspection that uses lighting to suppress the background from being extracted. This system could detect a variety of gold wire bonding defects in integrated circuit chips, such as broken, lost, shifted, or sagging bonding wires. However, this method is not suitable for use with complex wire bonding. Long et al. [15] developed a bonding joint inspection system based on image features and a support vector machine. Chen et al. [16] devised a data-driven method for wire bonding segmentation on X-ray images. In addition, they used CNNs to classify the segmentation results, with results superior to those obtained with traditional machine learning algorithms. Chan et al. [17] proposed a two-stage ball bonding detection method. First, the framework completes most of the ball bonding detection and classification tasks using CNNs and other methods. When the confidence level of the detection is below a certain threshold, experienced workers will perform manual inspection. Xie et al. [3] designed a learning-based algorithm to extract gold wire bonding structures from the an integrated circuit chip point cloud automatically. However, for complex integrated circuit chips, it is extremely difficult to obtain the required point cloud data. Overall, these vision-based detection methods perform well for wire bonding inspection, but they either require high-quality images or can only be adapted to a single scenario, such as bonding spot or gold wire detection. Moreover, these methods are not suitable for measuring bonding distances. To address the above issues, we establish a hierarchical measurement framework, including MWBINet and a BDMM, to simplify the distance measurement into bonding spot detection and gold wire segmentation using a visual detection method based on deep learning.

### 2.2. Learning-Based Object Detection

With the development of deep learning, object detection based on deep learning has become an important aspect of computer vision research [18,19,20,21,22,24]. CNN detectors, which are the most commonly used learning-based object detection algorithms, aim to find all objects of interest in an image and determine their categories and positions. Based on the steps used for object detection, these detectors can be divided into two main categories: two-stage object detectors and one-stage object detectors. The two-stage detectors use two steps consisting of region proposal and region-wise classification. A representative example of two-stage detectors is the “R-CNN family”, which includes R-CNN [18], Fast R-CNN [19], and Faster R-CNN [20]. The one-stage detector is a popular alternative, owing to its high efficiency and simplicity. The most popular one-stage detector is YOLO [21], which divides the image into many boxes using a grid and predicts bounding boxes for the object of interest in these boxes. Many improved methods have been developed based on the YOLO detector, such as YOLOv7 [25] and YOLOv8 [26], which have achieved good performance for object detection.

### 2.3. Learning-Based Semantic Segmentation

Similar to the learning-based object detection methods described above, state-of-the-art semantic segmentation approaches [10,11,27] are mainly based on CNNs. In an initial attempt to achieve semantic segmentation using CNNs, Jonathan Long [10] modified the contemporary classification network to the fully convolution network (FCN) for the semantic segmentation task. FCN performs end-to-end training and achieves pixel-level classification using intensive predictions. Yet, the segmentation accuracy of FCN is limited owing to the downsampling operation. In addition, FCN ignores potentially useful low-level feature information in previous feature maps. SegNet [28] is an encoder–decoder architecture based on modification of VGG16 [29] for semantic segmentation. This method introduces a maximum pooling index into the decoder, significantly improving the efficiency of segmentation. U-Net [11] introduces a skip connection into the backbone network, enabling the decoder to learn relevant features lost in the encoder by pooling at each stage. More recently, the DeepLab series [27,30,31,32] of algorithms proposed by Google researchers have shown outstanding performance. The DeepLabV1 [30] architecture mainly uses atrous convolution and a fully connected conditional random field (CRF). DeepLabV2 [32] adds atrous spatial pyramid pooling (ASPP), inspired by spatial pyramid pooling [33], using several atrous convolution modules with different rates to enhance the multi-scale recognition capability of the model. DeepLabV3 [31] reduces the resolution of feature maps by reconstructing networks based on DeepLabV2. The CRF module, which has been proved to be ineffective, was removed in DeepLabV3. DeepLabV3+ [27] introduces bilinear interpolation sampling in the decoder module, which improves the image semantic segmentation of the edge part.

## 3. Method

**Figure 2:**
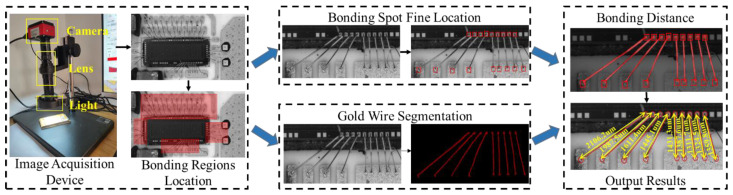
Overview of our hierarchical measurement structure for the integrated circuit bonding distance inspection task. First, the bonding regions are coarsely located by several convolution layers. Second, the bonding regions are fed into two parallel modules for bonding spot detection and gold wire segmentation. Third, the detected bonding spots are matched using the gold wire segmentation information in the BDMM. Last, the bonding distance is measured by extracting the center of each bonding spot and calculating the distance corresponding to the center coordinates. The output comprises the bonding distances of all wire bondings in the image.

We implement wire bonding distance inspection based on hierarchical measurement structure, as shown in Figure 2. The structure contains an image acquisition device, bonding regions location module, bonding spot fine location module, and gold wire segmentation module.

Among them, we design an image acquisition device to capture raw images from the surface of the integrated circuit with high resolution, which is the basic module used to achieve gold wire bonding defect inspection. There are three key components of this device: camera, high-magnification lens, and light, which are in charge of image collection, image zoom, and illumination supplement, respectively. Specifically, the camera model is HK830, manufactured by Jieshixin in Nanjing, China, and the output image resolution is 3840×2160. The model of the high-magnification lens is AO-HK830-5870, is also from Jieshixin, Nanjing, China. The height of the bracket is 355 mm, the focusing range is 65 mm, and the center range is 160 mm. Next, in this section, we first introduce the overall method, followed by a detailed description of the multi-branch wire bonding inspection network and the bonding distance measurement module.

### 3.1. Overview

As shown in Figure 1, gold wire bonding distance inspection is extremely challenging, as the gold wire is slender and the bonding spot is very small in the high-resolution images, which makes the location of the bonding difficult. In addition, the structure of the wire bonding is complex, which further increases the difficulty of the distance calculation. We design a hierarchical measurement structure that solves these problems.

First, we coarsely locate the bonding regions through several stacked convolutional layers. Since the gold wires on an integrated circuit board are usually clustered rather than discrete, we implement the coarse location to extract the bonding region. Thus, the features of the bonding spot and gold wires are amplified, and the accuracy of detection of the final location of the bonding is improved.

Subsequently, the extracted bonding region images are fed into the MWBINet with its fine location branch and wire segmentation branch to detect bonding spots and segment the gold wire. As the bonding spot is tiny and the gold wire is slender, it is difficult for general object detection methods [26,34,35] to locate them with high accuracy. To address this issue, we utilize spatial correlation information to improve the low positioning accuracy caused by the lack of pixel information. The two branches first extract low-level features via a shared backbone. Then, we separately employ the bidirectional feature fusion module and an edge module to extract the bonding spot and gold wire information. Given the spatial correlations between the bonding spots and gold wires, we utilize the semantic information in the segmentation branch as a semantic embedding to enhance the feature representation of the fine location branch.

Finally, we implement bonding spot center extraction and bonding spot matching for accurate bonding distance measurement.

### 3.2. Multi-Branch Wire Bonding Inspection Framework

**Figure 3:**
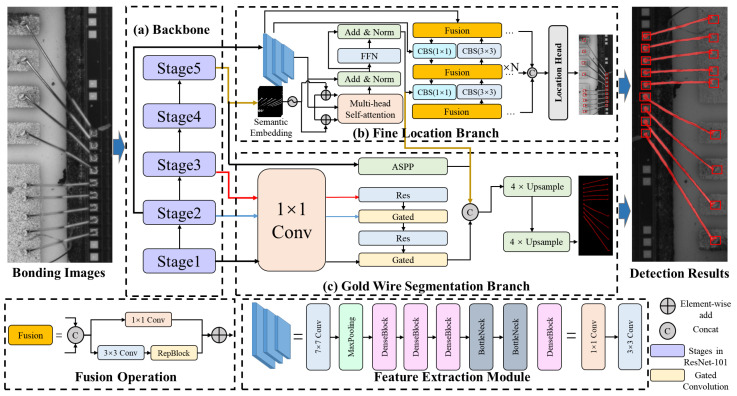
Structure of MWBINet, containing the fine location branch and the wire segmentation branch. The two branches are used to extract the bonding spot and gold wire information. The fine location branch consists of the feature extraction module, spatial correlation sensing module, and the bidirectional feature fusion module. The gold wire segmentation branch contains an edge module and an ASPP module.

In the hierarchical measurement structure, we apply MWBINet to accomplish the wire bonding location task initially, as shown in Figure 3. The fine location branch and the wire segmentation branch are separately applied to locate the bonding spot and segment the gold wire.

#### 3.2.1. Fine Location Branch

After the process of coarse bonding region localization, the extracted bonding region images are fed into the fine location branch and gold wire segmentation branch for the detection of the bonding spots and gold wire. As shown in Figure 1, all the bonding spots of integrated circuits are of small size, and the gold wire is slender, which means that they provide few features to the location model. Therefore, we design a multi-branch structure to perform localization and segmentation tasks. The set of information obtained by the two branches complements each other, enhancing the feature representation of each branch. Compared with other detection or segmentation models, our structure utilizes the unique spatial relationships between the solder joints and the golden wire. This improves the precision of positioning and segmentation.

The two branches first pass through a shared backbone and then extract the features of the gold wire and solder joint, respectively, on two parallel branches. In the fine location branch, we utilize a feature extraction module, a spatial correlation aware module, and a bidirectional feature fusion module to accomplish bonding spot location.

**Figure 4:**
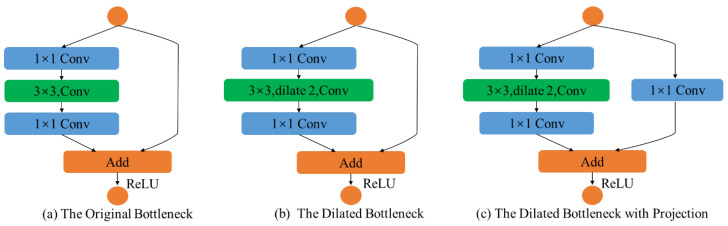
Structure of the bottleneck. The orange circles indicate the input and output of the bottlenecks. (**a**) The original bottleneck, which consists of 1×1 and 3×3 convolutions. (**b**) The dilated bottleneck, which consists of 1×1 and 3×3 dilated convolutions. (**c**) The dilated bottleneck with a 1×1 convolution projection.

Our feature extraction module is composed of three stages, which consist of various convolution layers, dense blocks, and bottlenecks. The first stage is a convolution layer with a kernel of 7×7, followed by 3×3 maxpooling layers and a dense block, containing alternations of 1×1 convolutions and 3×3 convolutions. The second stage contains two dense blocks. Since each layer of dense blocks is directly connected to the previous layer, the dense blocks can strengthen feature propagation and improve the utilization rate of feature maps. Meanwhile, since the **1×1** convolution in the dense block can reduce the dimension of the feature map, relatively small numbers of parameters in dense blocks can reduce the computational complexity of training. Finally, the last stage contains two dilated bottlenecks and one dilated bottleneck with a 1×1 convolution projection. The structure of the bottleneck is shown in Figure 4.

The spatial correlation sensing module is a single-layer transformer module. Since high-level features contain rich semantic information, we utilize a self-attention mechanism to obtain global information. The spatial correlation perception module placed after the feature extraction module can capture the correlation between high-level feature entities. Furthermore, the transformer block fuses the semantic information from the last stage of the backbone of the segmentation branch. The features from the segmentation branch are applied as a semantic embedding, which is added element-wise to the *Q* and *K* matrices in the self-attention calculation. The semantic embedding matrix strongly enhances the feature representation of the *Q* and *K* matrices, such that the feature attention will focus more on the spatial correlation of the bonding spot and the gold wire. The calculation process can be represented as follows:

(1) $$\mathrm{Atten}\left(Q,K,V\right)=\mathrm{softmax}\left(\frac{\left(Q⊕E\right){\left(K⊕E\right)}^{T}}{\sqrt{d_{k}}}\right)V,$$

where *Q*, *K*, and *V* are the query, key, and value information extracted from the feature extraction module, respectively. *E* represents the semantic embedding from the segmentation branch.

To improve the accuracy of bonding spot detection, single-level features should be extended to multi-level features by the feature fusion method, as in FPN [36]. On the downside, FPN only uses one-way feature fusion without weight, which will lead to unbalanced feature fusion. To solve this problem, we employ a bidirectional feature fusion module to realize variable-weighted feature fusion and a bidirectional feature flow.

The bidirectional feature fusion module has two major characteristics: bidirectional connections and variable-weighted feature fusion. First, the bidirectional connections overcome the limitations of conventional top-down FPN, which is a one-way information flow, thereby obtaining more information from different feature maps. In this paper, we implement bidirectional feature fusion through the repeated fusion operation. The fusion operation is realized by RepBlock and the convolution layers. Second, variable-weighted feature fusion balances the features from different layers, as follows:

(2) $$\begin{array}{c} O=\sum_{i}\frac{w_{i}}{\varepsilon +\sum_{j}w_{j}}\cdot I_{i}, \end{array}$$

where $w_{i}\geq 0$ represents the variable weight guaranteed, ε is a parameter to avoid a zero denominator, and $I_{i}$ represents a feature from the *i*-th layer.

#### 3.2.2. Gold Wire Segmentation Branch

The gold wire segmentation branch functions in parallel with the bonding spot location branch. It aims to obtain an explicit and continuous gold wire edge with high segmentation precision. Its main components are an edge module and an ASPP [32] module. In addition, the bonding spot location branch provides spatial correlation information as auxiliary information to enhance the representation of gold wire features.

First, the input of the segmentation branch is $I\in R^{3\times H\times W}$, where *I*, *W*, and *H* represent the image and its width and height, respectively. We denote the output feature representation of the backbone as $r\in R^{C\times \frac{H}{m}\times \frac{W}{m}}$, where *m* is the stride of the backbone and *C* is the number of channels of the backbone network.

Parallel to the backbone network, the edge module contains several residual blocks and GCLs. The edge module takes the output of the backbone network’s first three stages as input. Specifically, as the edge module contains two GCLs, it can be divided into two layers. The feature map of the edge module is $s\in R^{H\times W}$. In addition, the GCL ensures that the edge module only processes edge-relevant information. Finally, the ASPP module fuses features from different layers with different resolutions; this step has been widely used in semantic segmentation algorithms.

Our segmentation branch can also be regarded as an encoder–decoder structure. Specifically, the encoder module consists of the backbone network, the edge module, and the ASPP module. The outputs of the encoder module are regular feature maps from the ASPP module and low-level edge feature maps from the edge module. In the decoder module, we reconstruct the original segmentation map step by step. First, we upsample the regular feature maps based on a bilinear interpolation scheme. Then, the regular feature maps from ASPP are fused with the low-level edge feature maps from the edge module and the auxiliary feature from the fine location branch. Finally, we perform a 3 × 3 convolution and an upsampling on the feature map to produce the final segmentation output.

**Gated Convolutional Layer.** GCL is the core component of the segmentation branch, which extracts only edge-relevant information by filtering out other information from the regular feature maps of the backbone. Specifically, the operation of the GCL can be divided into two steps. First, we obtain an attention map from the feature maps $r_{t}$ and $s_{t}$:

(3) $$\begin{array}{c} \alpha_{t}=\sigma \left(C_{1\times 1}\left(s_{t}\|r_{t}\right)\right), \end{array}$$

where $r_{t}$ and $s_{t}$ denote the feature maps of the backbone and edge modules, respectively; $C_{1\times 1}$ represents a 1×1 convolutional layer; ‖ denotes feature concat; σ is the sigmoid function; and $\alpha_{t}\in R^{H\times W}$ is the output attention map.

Next, we apply the element-wise product ⊙ on $s_{t}$ with attention map α, followed by a residual connection. We define the GCL as:

(4) $$\begin{array}{c} {\hat{s}}_{t}^{\left(i,j\right)}={\left(s_{t}⊛\omega_{t}\right)}_{\left(i,j\right)}={\left(\left(s_{t_{\left(i,j\right)}}⊙\alpha_{t_{\left(i,j\right)}}\right)+s_{t_{\left(i,j\right)}}\right)}^{T}\omega_{t}, \end{array}$$

where ⊙ represents the element-wise product, and ${\hat{s}}_{t}$ is the output low-level edge feature map of the edge branch. Intuitively, α denotes an attention map with more edge information. We have two GCLs that connect to the first three stages of the backbone.

**Gold Wire Segmentation Loss (GWSL) Definition.** In the segmentation branch, we jointly supervise segmentation and edge feature map prediction. Specifically, we design a GWSL function with four parts. The first two parts are the semantic segmentation loss ($L_{ss}$) and the edge loss ($L_{e}$). We use the standard cross-entropy (CE) loss and standard binary cross-entropy (BCE) loss on predicted semantic segmentation *f* and edge feature maps *s*, respectively. The definitions for $L_{ss}$ and $L_{e}$ can be described as:

(5) $$\begin{array}{c} L_{ss}=\lambda_{1}L_{CE}\left(\hat{y},f\right), \end{array}$$

(6) $$\begin{array}{c} L_{e}=\lambda_{2}L_{BCE}\left(s,\hat{s}\right), \end{array}$$

where $\hat{y}$ and $\hat{s}$ represent ground truth (GT) labels, and $\lambda_{1}$, $\lambda_{2}$ are two balancing parameters; $\lambda_{1},\lambda_{2}\in \left[0,1\right]$. Specifically, the balancing parameters are set to balance the influence of regular information and edge information in the detection process.

In addition, to prevent overfitting caused by foreground–background class imbalance, we propose two regularization loss functions, which constitute the other two parts of GWSL. We utilize the first regularization loss ($L_{r1}$) to avoid GWSL offset due to mismatching between the GT edge and the predicted edge. The $L_{r1}$ can be defined as:

(7) $$\begin{array}{c} L_{r1}=\lambda_{3}\sum_{p^{+}}|\zeta (p^{+})-\hat{\zeta }\left(p^{+}\right)|, \end{array}$$

where ζ is a confidence value indicating whether a pixel belongs to the gold wire edge, $\hat{\zeta }$ is a similar value computed from the GT, and $p^{+}$ represents the set of predicted pixel coordinates.

Specifically, the value ζ is computed as:

(8) $$\begin{array}{c} \zeta =\frac{1}{\sqrt{2}}\|\nabla \left(G*\underset{k}{\arg\max}p\left(y^{k}|r,s\right)\right)\|, \end{array}$$

where *G* is the Gaussian filter, and $p(y^{k}|r,s)$ is a label distribution of the prediction.

Moreover, we implement edge prediction to match semantic prediction, which also prevents overfitting:

(9) $$\begin{array}{c} L_{r2}=\lambda_{4}\sum_{k,p}{⊮}_{s_{p}}\left[{\hat{y}}_{p}^{k}\log p(y_{p}^{k}|r,s)\right], \end{array}$$

where *p* and *k* represent the set of pixels and the set of labels, respectively; ${⊮}_{s_{p}}=\left\{1:s>thrs\right\}$ denotes the indicator function; thrs denotes a threshold, which we set to 0.8 in the segmentation branch; and $\lambda_{3}$ and $\lambda_{4}$ denote two balancing parameters. Specifically, we set $\lambda_{3}=0.15$ and $\lambda_{4}=0.11$ in our experiments to optimize the performance of our segmentation branch.

### 3.3. Bonding Distance Measurement Module

**Figure 5:**
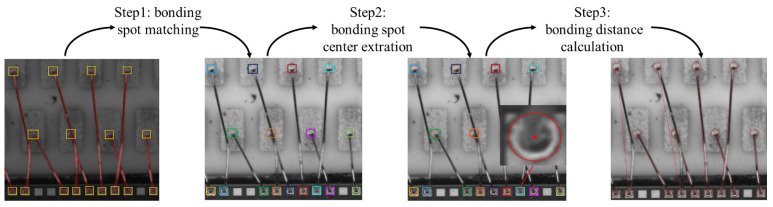
Pipeline of the bonding distance measurement module, which consists of three steps: (1) bonding spot matching; (2) bonding spot center extraction; (3) bonding distance calculation. Different colors indicate different detection box for bonding spots.

The bonding distance is defined as the projected distance between the centers of two corresponding bonding spots. Therefore, we need to find the corresponding bonding spots first. Nonetheless, given the complex distribution of gold wire bondings, it is difficult to directly calculate the bonding distance. To address this issue, we design a BDMM to gradually calculate the bonding distance based on bonding spot detection and gold wire segmentation results, as shown in Figure 5.

Specifically, the bonding distance measurement has three main steps. First, the bonding spots are matched based on the gold wire segmentation results. Next, the center of each bonding spot is extracted using a RANSAC-based method. Finally, the bonding distance—that is, the distance between two corresponding boding spots—is confirmed based on the identified center coordinates.

**Figure 6:**
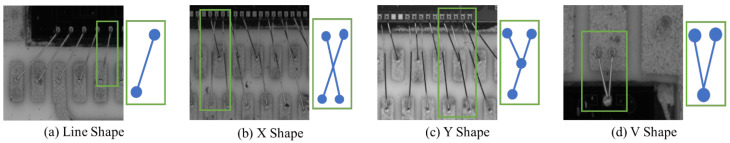
Four gold wire bonding distribution models: (**a**) line shape, (**b**) X shape, (**c**) Y shape, (**d**) V shape.

**Step 1 (bonding spot matching):** For an integrated circuit image containing many detected bonding spots, we first need to match the corresponding two bonding spots connected by the gold wire. In fact, observation of the segmentation results for gold wire bonding shows that the distribution of the gold wire is very complicated. As a result, it is not possible to match bonding spots directly by nearby screening. As shown in Figure 6, based on the analysis of the segmentation results for gold wire, we establish four gold wire distribution models, representing straight-line, X, Y, and V shapes. Specifically, we first classify each segmented and connected region according to the gold wire distribution model. Furthermore, the bonding spots near the end of each segmented gold wire are searched and matched. We design different neighborhood search rules for different distribution types. Finally, the matched corresponding bonding spots are represented by the bounding box of the same color, as shown in Figure 5.

**Step 2 (bonding spot center extraction):** Based on the definition of the gold wire bonding distance, we need to extract the bonding spot center. The areas of the detected bonding spots in the fine location branch are cropped into several small regions. As the bonding spot is not a standard circle, it cannot be fitted by Hough circle detection. Therefore, we use a RANSAC-based circle fitting method to extract the bonding spot circle, considering RANSAC-based method finds the center of the circle by randomly selecting a minimal subset of the data points and using the points to fit the circle. This method is not only suitable for extracting the center of ball bonding, but also suitable for extracting the center of wedge bonding.

**Step 3 (bonding distance measurement):** Having determined the relations of the bonding spots and all the bonding spot centers through the above two steps, we denote the centers of the corresponding bonding spots by $P_{1}$ and $P_{2}$. The pixel distance of wire bonding is computed as follows:

(10) $$\begin{array}{c} D_{P}=\sqrt{{\left(x_{P_{1}}-x_{P_{2}}\right)}^{2}+{\left(y_{P_{1}}-y_{P_{2}}\right)}^{2}}, \end{array}$$

where $D_{P}$ represents the pixel distance of the wire bonding; $x_{P_{1}}$ and $y_{P_{1}}$ represent the horizontal and vertical coordinates of $P_{1}$ in the image; and $x_{P_{2}}$ and $y_{P_{2}}$ represent the horizontal and vertical coordinates of $P_{2}$ in the image, respectively.

We calibrate the camera in advance to measure the physical distance of each pixel, so pixel distance can be easily converted into physical distance. Specifically, the internal parameter matrix, external parameter matrix, and distortion coefficients of the camera are solved using the Zhang Zhengyou plane calibration method. Then, we determine the proportional relationship between the object and the pixel by measuring the standard block. Therefore, the bonding distance can be calculated as:

(11) $$\begin{array}{c} \frac{W}{N}=K, \end{array}$$

where *W* represents the physical size of the standard measurement block, *N* represents the pixel size of the standard measurement block in the image, and *K* represents the equivalent pixel unit. Industrial cameras capture images at the same height, and the corresponding physical size per pixel does not change. Subsequent experiments have shown that this method can be used to measure the bonding distance efficiently and accurately.

## 4. Experiments and Results

### 4.1. Experimental Settings

We introduce three types of datasets, corresponding to the three types of detected objects: the bonding region, bonding spot, and gold wire. Raw images with high resolution are captured from the surface of the integrated circuit by the image acquisition device. First, the bonding region of the raw images is roughly marked for training of the bonding region location branch. After training, this branch can detect and extract the bonding regions and filter out the complex background. Next, the bonding spots and gold wires are extracted from the bonding region images for fine labeling manually. Finally, we divide the three annotated datasets into a training set, a validation set, and a testing set. Specifically, the image datasets are captured from 200 integrated circuits and include 1000 images. We use a series of image enhancement techniques to expand the 1000 images to 10,000 images, including rotation, translation, brightness adjustment, and adding random noise. All subsequent training was conducted based on these augmented 10,000 images. The proportions of the training set, validation set, and test set are 80%, 10%, and 10%, respectively. Moreover, the bonding spot dataset and the gold wire dataset consist of the bonding region images extracted from the bonding region dataset.

During training, we use Pytorch and train the network on an NVIDIA GTX 3090 GPU from Jieshixin in Nanjing, China. The network is trained with the AdamW optimizer with a batch size of 16 and an initial learning rate of 0.001 decreased by 0.6 at every 20 epochs. The solver is a standard stochastic gradient descent with a momentum of 0.9.

### 4.2. Comparisons

**Comparison of fine location branch with state-of-the-art methods.** To validate the performance of the proposed fine location branch, we compare our method with state-of-the-art object detection methods, including RetinaNet [37], CenterNet [38], EfficientDet [35], Sparse-RCNN [34], RT-DETR [39], YOLOv8 [26], and Salience DETR [40], as shown in Table 1. The fine location branch achieves accuracy, precision, recall, and F-measure rates of 94.5%, 93.0%, 94.8%, and 93.9%, respectively. Notably, the accuracy of our method is 4.9% higher than that of Salience DETR and 16% higher than that of RT-DETR, indicating significant improvement. This enhancement is attributed to the limitations of models such as RetinaNet, CenterNet, Sparse-RCNN, YOLOv8, and Transformer models (e.g., Salience DETR and RT-DETR) in detecting small objects, as the multi-layer self-attention mechanism often leads to the loss of information about small objects. Additionally, EfficientDet shows performance declines in highly complex or dense scenes, such as integrated circuit bonding distance inspection, which involves numerous dense bounding spots.

**Figure 7:**
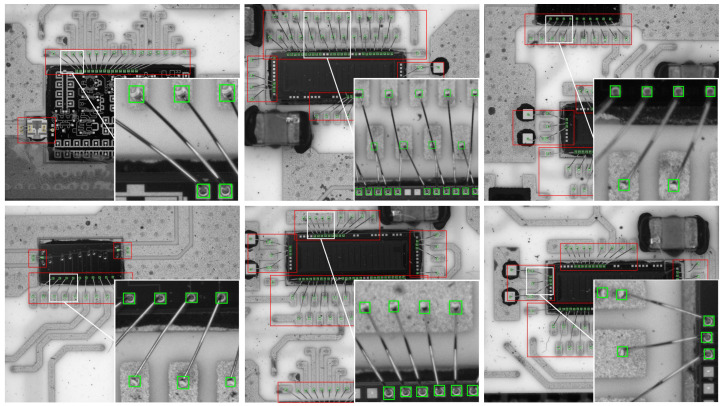
Some detection results for tiny bonding spots. Red boxes indicate the bonding region from the coarse location; green boxes indicate the bonding spots detected by the fine location branch.

Our proposed feature extraction module addresses these challenges by using dense blocks and dilated convolutions, enhancing the detection of small bonding spot targets. Furthermore, the bidirectional feature fusion module improves bonding spot detection by integrating information from different feature maps. Some detection results are shown in Figure 7.

**Figure 8:**
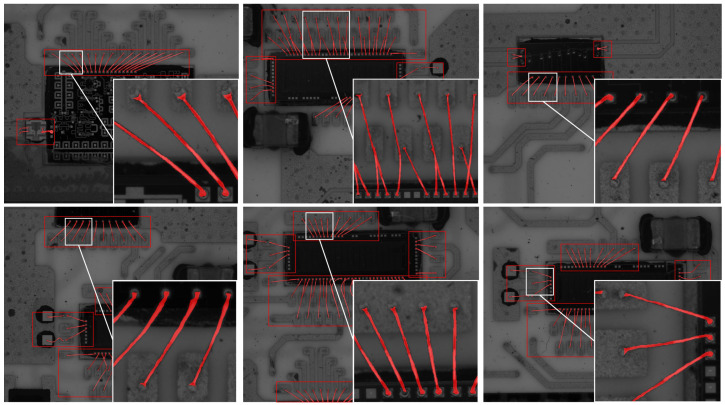
Some segmentation results for gold wires. Red boxes indicate the bonding region from the coarse location; red masks indicate the gold wire.

**Comparison of segmentation branch with state-of-the-art methods.** Finally, we compare our gold wire segmentation branch with state-of-the-art segmentation methods, including FCN [20], U-Net [11], Seg-Net [28], PSP-Net [41], deeplabv3+ [27], SeaFormer [42], SAM [43], SSA [44], and Rein [45]. As shown in Figure 8, which provides a visualization of the results, in the gold wire segmentation by FCN, U-Net, Seg-Net, and PSP-net, the single gold wire segmentation is disconnected, resulting in a poor segmentation effect. DeepLabv3+ and our algorithm achieve complete segmentation of a single gold wire, but with the DeepLabv3+ algorithm, the segmentation width is quite different from the ground truth in some parts. The reason is that FCN, U-Net, Seg-Net, and PSP-Net, the early semantic segmentation networks, are not good at dealing with small targets like gold wires. The features of gold wires are easily lost during the downsampling and upsampling processes. Although DeepLabv3+ utilizes atrous convolution to enhance performance, the improvement is limited, and some small-sized features are still overlooked.

As shown in Table 2, since our segmentation branch can process edge features separately via the edge branch to improve the segmentation accuracy for gold wire bonding, the results of our segmentation branch are superior to those of the other networks in terms of mean pixel accuracy (MPA) and MPA (86.2%) and mean intersection over union MIOU (87.3%). Specifically, our MIOU is 1.4% higher than the MIOU of the state-of-the-art Rein method and 2.5% higher than the well-known SAM. Figure 8 shows some examples of gold wire segmentation results obtained with our method.

### 4.3. Evaluation of Bonding Distance Measurement

The goal of this study is to measure bonding distances. Therefore, we verify the effectiveness of the whole framework on real integrated circuit components.

First, we calibrate the pixel equivalent unit based on the standard block. The size of the standard block was 1000 μm, and we calculated the average pixel size of the standard block to be 232.76 pixels based on eight measurements. The pixel equivalent unit is computed as:

(12) $$\begin{array}{c} K=\frac{W}{N}=\frac{1000}{232.76}\left(\mu m/\mathrm{pixel}\right)=4.3\left(\mu m/\mathrm{pixel}\right), \end{array}$$

where *W* represents the physical size of the standard measure block, *N* represents the pixel size of the standard measure block in the image, and *K* represents the pixel equivalent unit.

Second, we compare our method with microscopy-based methods on 20 integrated circuit components. Specifically, the average processing time of our method for an image is 0.64 s. The average processing time of our method for an integrated circuit component is about 10 s, much shorter than that of microscopy-based methods. In addition, our method is automated and does not require manual operation.

**Figure 9:**
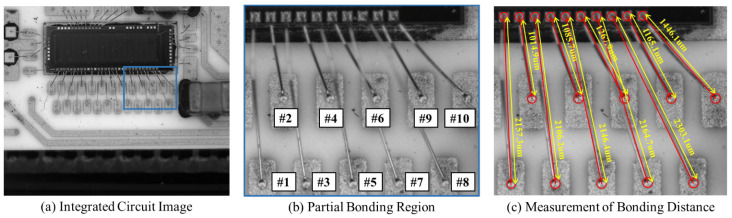
An integrated circuit image was used to evaluate the bonding distance measurement method. (**a**) Integrated circuit image. (**b**) Partial bonding region. “#1–#10” denotes ten gold wires numbered from 1 to 10. (**c**) Measurement of bonding distance.

Finally, we verify the effectiveness of the BDMM on the integrated circuit shown in Figure 9. The bonding distance GT for the gold wires of this integrated circuit is measured using a microscope. We select ten wire bondings from the input integrated circuit image for the experiment. To improve the robustness of the measurement, we perform 20 measurements and took the average of these as the final bonding distance. Table 3 shows the bonding distance results for ten gold wire bondings, including GT bonding distance, mean value bonding distance, mean error, standard deviation, and maximum error. Our method achieves values of 8.3 μm, 12.5 μm, and 0.56% for mean error, maximum error, and relative error; the mean errors of the 20 measurements of the wire bonding distance were between −10.4 and 11.3 μm. These results demonstrate that the BDMM can achieve a high-precision bonding distance measurement.

### 4.4. Ablation of Key Component Modules

The ablation results for each component module are presented in Table 4. It is evident that the absence of the coarse location branch significantly diminishes its performance, with precision decreasing by 11.2% and the mean error reaching 42.3%.

In the segmentation branch, the omission of the semantic embedding and bidirectional feature fusion module resulted in suboptimal extraction of information related to the bonding spot and gold wire, owing to the neglect of the spatial correlation between them. As a result, the precision, recall, and F-measurement are decreased, with MPA and MIOU experiencing a slight decline. Specifically, the precision of MWBINet without semantic embedding was 90.1%, which is 2.9% lower than that of MWBINet with semantic embedding.

In the location branch, when the edge branch and GWSL were not utilized, overfitting caused by foreground–background class imbalance may occur. Thus, the performance of segmentation becomes less effective to a certain extent. The MPA and MIOU of MWBINet without the GSWL register show declines of 5.1% and 5.5%, respectively.

In conclusion, employing all five modules simultaneously enhances precision and maintains high MPA and low relative error. This integrated approach is pivotal for achieving accurate and efficient bonding distance measurements.

## 5. Conclusions and Future Work

In this paper, we systematically present a framework for measuring bonding distances in integrated circuit images. First, we use coarse localization to remove redundant information from the integrated circuit images. Next, we implement the fine localization branch and the gold wire segmentation branch to detect bonding spots and segment the gold wire. Finally, we apply a bonding distance measurement module to connect the corresponding bonding spots and calculate the bonding distance between them. Experimental results show that the proposed bonding distance measurement framework achieves a stable and efficient bonding distance measurement. In addition, our model can be applied in other industrial quality inspection tasks, such as pipe crack detection and metal surface scratch detection, evaluating the quality of the inspection object through distance measurement.

In the present work, we have not taken into account production defects in gold wire bonding other than abnormal bonding distances. If other manufacturing defects are present in gold wire bonding, measuring the bonding distance of the defective bonds will be meaningless; therefore, we must remove the gold wire bonds with other defects first. Unfortunately, due to the low frequency of production defects in gold wire bonding, we were unable to collect enough defect images for training. In the future, we will conduct bonding defect detection to expand the range of indicators in our bonding inspection and measurement framework, and we will continue to expand the application scenarios of our model.

## Figures and Tables

**Table 1:** Results of the detection of bonding spots from integrated circuit images.

Method	Years	Accuracy (%)	Precision (%)	Recall (%)	F-Measure (%)
RetinaNet	2017 [37]	88.7	86.3	85.2	87.5
CenterNet	2019 [38]	90.6	89.5	89.2	89.3
EfficientDet [35]	2020 [35]	90.4	91.2	89.9	90.5
Sparse-RCNN	2021 [34]	84.5	86.2	87.3	86.7
RT-DETR	2023 [39]	78.5	73.7	76.2	77.3
YOLOv8	2023 [26]	83.7	82.5	86.5	84.4
Salience DETR	2024 [40]	89.6	87.7	91.3	86.8
Our method	-	**94.5**	**93.0**	**94.8**	**93.9**

Bolded data indicate the best results for the same indicator.

**Table 2:** Comparison of gold wire segmentation results from integrated circuit images.

Method	Years	MPA (%)	MIOU (%)
FCN	2015 [20]	73.6	70.2
U-Net	2015 [11]	75.6	79.3
Seg-Net	2017 [28]	71.5	79.2
PSP-Net	2017 [41]	79.1	80.9
DeepLabv3+	2018 [27]	84.3	83.1
SeaFormer	2023 [42]	85.2	81.7
SAM	2023 [43]	85.8	84.8
SSA	2024 [44]	84.5	86.3
Rein	2024 [45]	85.2	85.9
Our method	-	**86.2**	**87.3**

Bolded data indicate the best results for the same indicator.

**Table 3:** Bonding distance measurement results for real integrated circuits.

Gold Wire	GT	Mean Value (μm)	Mean Error (μm)	Maximum Error (μm)	Relative Error (%)
#1	2147.9	2157.3	9.4	13.5	0.44
#2	1003.6	1014.9	11.3	14.5	1.13
#3	2110.6	2106.2	−4.4	−9	0.21
#4	1077.9	1085.7	7.8	11.6	0.72
#5	2140.1	2146.4	6.3	10.1	0.29
#6	1258.1	1267.9	9.8	15.1	0.78
#7	2175.1	2164.7	−10.4	−14.3	0.48
#8	2294.8	2303.1	8.3	13.6	0.36
#9	1158.2	1165.1	6.9	10.6	0.60
#10	1454.6	1446.1	−8.5	−13.1	0.59
Average	-	-	8.3	12.5	0.56

**Table 4:** Ablation of key component modules.

Method Error (%)	Precision (%)	Recall (%)	F-Measure (%)	MPA (%)	MIOU (%)	Mean Error (%)	Relative
MWBINet w/o CL	81.2	80.6	80.3	74.3	71.2	42.3	6.7
MWBINet w/o SE	90.1	89.7	90.6	86.1	87.1	9.1	0.59
MWBINet w/o BFFM	89.3	89.6	90.3	86.0	87.0	9.3	0.62
MWBINet w/o EB	90.3	90.4	88.7	83.2	82.9	12.5	0.89
MWBINet w/o GSWL	90.1	90.2	89.3	81.1	81.8	13.6	0.94
MWBINet	**93.0**	**94.8**	**93.9**	**86.2**	**87.3**	**8.3**	**0.56**

Bolded data indicate the best results for the same indicator. SE: semantic embedding. BFFM: bidirectional feature fusion module. EB: edge branch. GSWL: gold wire segmentation loss. MWBINet: multi-branch wire bonding inspection network. CL: coarse location. w/o: without.

## Data Availability

Data available on request due to restrictions. The data presented in this study are available on request from the corresponding author due to the commercial confidentiality involved.
